# A Practical and Time-Efficient High-Intensity Interval Training Program Modifies Cardio-Metabolic Risk Factors in Adults with Risk Factors for Type II Diabetes

**DOI:** 10.3389/fendo.2017.00229

**Published:** 2017-09-08

**Authors:** Bethan E. Phillips, Benjamin M. Kelly, Mats Lilja, Jesús Gustavo Ponce-González, Robert J. Brogan, David L. Morris, Thomas Gustafsson, William E. Kraus, Philip J. Atherton, Niels B. J. Vollaard, Olav Rooyackers, James A. Timmons

**Affiliations:** ^1^Clinical, Metabolic and Molecular Physiology Research Group, School of Medicine, University of Nottingham, Derby, United Kingdom; ^2^Nuffield Health, Epsom, United Kingdom; ^3^Department of Laboratory Medicine, Karolinska University Hospital, Stockholm, Sweden; ^4^Department of Physical Education, University of Las Palmas de Gran Canaria, Las Palmas de Gran Canaria, Spain; ^5^Scion House, Stirling University Innovation Park, Stirling, United Kingdom; ^6^Division of Genetics and Molecular Medicine, King’s College London, London, United Kingdom; ^7^Duke Molecular Physiology Institute, Duke University School of Medicine, Durham, NC, United States; ^8^Faculty of Health Sciences and Sport, University of Stirling, Stirling, United Kingdom; ^9^CLINTEC, Karolinska Institutet, Karolinska University Hospital, Stockholm, Sweden

**Keywords:** health, exercise, high-intensity interval training, variability, ${\dot{\text{V}}\text{O}}_{\text{2}}\text{ max}$, blood pressure, detraining, homeostatic model assessment of insulin resistance

## Abstract

**Introduction:**

Regular physical activity (PA) can reduce the risk of developing type 2 diabetes, but adherence to time-orientated (150 min week^−1^ or more) PA guidelines is very poor. A practical and time-efficient PA regime that was equally efficacious at controlling risk factors for cardio-metabolic disease is one solution to this problem. Herein, we evaluate a new time-efficient and genuinely practical high-intensity interval training (HIT) protocol in men and women with pre-existing risk factors for type 2 diabetes.

**Materials and methods:**

One hundred eighty-nine sedentary women (*n* = 101) and men (*n* = 88) with impaired glucose tolerance and/or a body mass index >27 kg m^−2^ [mean (range) age: 36 (18–53) years] participated in this multi-center study. Each completed a fully supervised 6-week HIT protocol at work-loads equivalent to ~100 or ~125% ${\dot{\text{V}}\text{O}}_{\text{2}}\text{ max}$. Change in ${\dot{\text{V}}\text{O}}_{\text{2}}\text{ max}$ was used to monitor protocol efficacy, while Actiheart™ monitors were used to determine PA during four, weeklong, periods. Mean arterial (blood) pressure (MAP) and fasting insulin resistance [homeostatic model assessment (HOMA)-IR] represent key health biomarker outcomes.

**Results:**

The higher intensity bouts (~125% ${\dot{\text{V}}\text{O}}_{\text{2}}\text{ max}$) used during a 5-by-1 min HIT protocol resulted in a robust increase in ${\dot{\text{V}}\text{O}}_{\text{2}}\text{ max}$ (136 participants, +10.0%, *p* < 0.001; large size effect). 5-by-1 HIT reduced MAP (~3%; *p* < 0.001) and HOMA-IR (~16%; *p* < 0.01). Physiological responses were similar in men and women while a sizeable proportion of the training-induced changes in ${\dot{\text{V}}\text{O}}_{\text{2}}\text{ max}$, MAP, and HOMA-IR was retained 3 weeks after cessation of training. The supervised HIT sessions accounted for the entire quantifiable increase in PA, and this equated to 400 metabolic equivalent (MET) min week^−1^. Meta-analysis indicated that 5-by-1 HIT matched the efficacy and variability of a time-consuming 30-week PA program on ${\dot{\text{V}}\text{O}}_{\text{2}}\text{ max}$, MAP, and HOMA-IR.

**Conclusion:**

With a total time-commitment of <15 min per session and reliance on a practical ergometer protocol, 5-by-1 HIT offers a new solution to modulate cardio-metabolic risk factors in adults with pre-existing risk factors for type 2 diabetes while approximately meeting the MET min week^−1^ PA guidelines. Long-term randomized controlled studies will be required to quantify the ability for 5-by-1 HIT to reduce the incidence of type 2 diabetes, while strategies are required to harmonize the adaptations to exercise across individuals.

## Introduction

Substantial correlative evidence indicates that exercise capacity and greater self-reported physical activity (PA) (1) both positively relate to health. In fact, aerobic capacity $\left({\dot{\text{V}}\text{O}}_{\text{2}}\text{ max}\right)$ measured in the laboratory appears to be a better predictor of health status and risk of disease than many other risk factors (2). Furthermore, guidance aimed at concurrently improving diet and increasing levels of PA has successfully demonstrated substantial reductions in the incidence or progression-rates of type 2 diabetes after 10 years of follow-up (3–5). It is currently a (reasonable) assumption that the increased levels of PA in these trials (3–5) made a major contribution to the improved metabolic health. Shorter-term exercise training intervention studies (6 weeks–6 months) attempt to quantify the physiological responses to exercise, relying on surrogates or “biomarkers” of health to explore the potential efficacy of very divergent training programs. These studies typically observe gains in aerobic capacity (6) and reductions in blood pressure (7) and insulin resistance (IR) following 6–40 weeks of supervised training (8). The format of each exercise training program (time and exercise intensity) have reflected PA guidelines developed from epidemiological observations, e.g., high-volume continuous submaximal aerobic training carried out on 3–5 days each week (7, 9) with the aim of meeting a time-commitment for voluntary exercise of 150 min week^−1^.

Studies using lower volume very high-intensity interval training (HIT) and highly specialist cycle ergometers have demonstrated that modulation of risk factors for type 2 diabetes can be achieved by exercising a total of 70–90 min week^−1^ in small groups of individuals (10–14). Nevertheless, while the total time for the “bouts” of exercise can be very low (≤5 min day^−1^), these formats of HIT require long recovery periods between each bout such that they do not substantially reduce the total time-commitment to a level that might substantially improve exercise participation. Some investigators have raised the possibility of gender-specific benefits, which most likely reflect the large amount of inter-individual variability observed in any exercise training study (15–17) and the small number of subjects studied when evaluating any particular variant of HIT (10–14, 18). The reliance on a wide range of HIT protocols has meant that neither the effect size nor the inter-individual variability has been properly quantified (10–14, 18) and such divergent protocols limit the validity of any meta-analysis approach to address these important questions. Indeed, the design of future large-scale outcome studies of novel exercise paradigms requires reliable estimates of the effect size in target at-risk populations and this study evaluated a more time-efficient protocol that overcomes some of the practical limitations of earlier studies. The initial HIT protocol was based on a 1981 study by Ready et al. (19). While the present project was not a randomized clinical trial, we did embrace the multi-arm multi-stage clinical trial *philosophy* (20), whereby we monitored the HIT protocol efficacy on a rolling basis, by aggregating the ${\dot{\text{V}}\text{O}}_{\text{2}}\text{ max}$training responses as we went along. This resulted in us discontinuing a 7-by-1 min HIT protocol (~100% ${\dot{\text{V}}\text{O}}_{\text{2}}\text{ max}$ cycling intensity), in favor of a lower volume, higher intensity protocol (5-by-1 min HIT, at ~125% ${\dot{\text{V}}\text{O}}_{\text{2}}\text{ max}$ cycling intensity). We were able to confirm that a practical and time-efficient 5-by-1 HIT protocol not only improved ${\dot{\text{V}}\text{O}}_{\text{2}}\text{ max}$ (on average), but also that this particular time-efficient exercise regime was equally effective in both men and women at modifying cardio-metabolic disease risk factors.

## Materials and Methods

The experimental design for the 7-by-1 HIT protocol and clinical testing procedures were discussed at a work-shop, in Las Palmas on January 30, 2012, with the following people, in addition to authors, in attendance: Martin Gibala, Jorn Helge, Fleming Dela, Ruth Loos, Laurie Goodyear, Claude Bouchard, Tuomo Rankinen, Jose Calbet, Urho Kujala, Heikki Kainulainen, Steen Larsen, Lauren Koch, and Paul Greenhaff.

### Participant Characteristics

**Figure 1:**
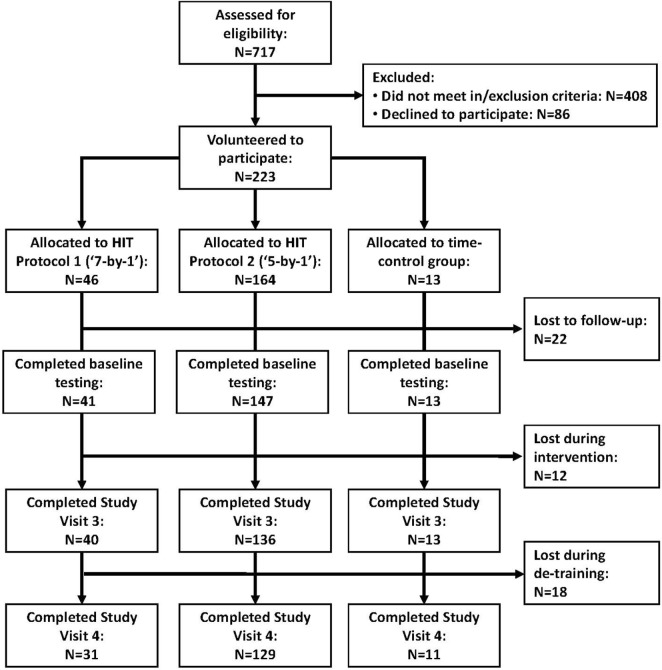
Flow chart of participant contact, screening, and retention through the phases of the study.

For the METAPREDICT HIT trial, we recruited 189 participants (Figure 1) across 5 geographical regions: Nottingham (*n* = 37) and Loughborough (*n* = 18) in the UK, Stockholm (Sweden, *n* = 36), Copenhagen (Denmark, *n* = 48), and Las Palmas de Gran Canaria (Spain, *n* = 50). All methods relied on across-site standard operating procedures. Participants were recruited *via* advertisements in local media, through publicity on the EU and University websites, and *via* links with radio and TV stations. We also used demographic databases to post information to potential volunteers and put out adverts in local community groups, particularly those involving sedentary adults. Participants were male (*n* = 88) and female (*n* = 101), with a mean (range) age of 36 (18–53) years and body mass index (BMI) of 32.0 (26.6–48.0) kg m^−2^. All participants were classified as sedentary [<600 metabolic equivalents (METs) min week^−1^] using a modified International Physical Activity Questionnaire (21), and had a fasting blood glucose level consistent with World Health Organization criteria for impaired glucose tolerance (IFG; >5.5, <7.0 mmol l^−1^), and/or a BMI > 27 kg m^−2^.

All participants were initially screened and excluded if they displayed evidence of active cardiovascular, cerebrovascular, respiratory, gastrointestinal, or renal disease. They were also excluded for history of malignancy, coagulation dysfunction, musculoskeletal or neurological disorders, recent steroid or hormone replacement therapy, or any condition requiring long-term drug prescriptions. All participants gave their written, informed consent to participate. This study was approved by local ethics committees at all sites (the University of Nottingham Medical School Ethics Committee: D8122011 BMS; the Regional Ethical Review Board Stockholm: 2012/753-31/2; the ethics committee of the municipality of Copenhagen and Frederiksberg in Denmark: H-3-2012-024; Comité Ético de Investigación Humana de la ULPGC: CEIH-2012-02; and the Loughborough University Ethics Approvals Human Participants Sub-Committee: 12/EM/0223) and complied with the 2008 Declaration of Helsinki. To ensure accurate results, we were obliged to discontinue training for individuals who (i) failed to attend for more than two consecutive sessions, (ii) missed more than three (~15%) training sessions in total, or (iii) failed to complete their set exercise regime on two occasions or more. This was not the case for any participants.

### HIT—Protocol 1 (“7-by-1”)

Forty participants [*n* = 20 men/20 women; age: 37 (20–53) years; BMI: 31.0 (27.0–45.5) kg m^−2^] completed a 7-by-1 HIT protocol (Table 1) developed using information from the literature (19, 22). 7-by-1 HIT protocol consisted of three fully supervised cycling sessions per week for 6 weeks. Sessions began with a 2-min warm-up at 50 W followed by seven sets of 1 min cycling at 100% of the work required to elicit ${\dot{\text{V}}\text{O}}_{\text{2}}\text{ max}$ (Corival or Excalibur Sport, Lode, Groningen, the Netherlands) with 1 min recovery between bouts. For 1 h before, during, and for 1 h after each training session, the participants were only allowed to consume water. No adverse events or unintended effects were observed with this intervention. However, based on interim analysis, 7-by-1 HIT was found to result in a relatively modest increase in ${\dot{\text{V}}\text{O}}_{\text{2}}\text{ max}$ (+6.2%) and, thus, was insufficient to assess inter-individual variability in response to training [SD of individual responses (SD_IR_): 106 mL O_2_; 95% CI: −6 to 218 mL (see Data Processing and Statistical Analysis)]. Reliance on a 25-W stepwise ${\dot{\text{V}}\text{O}}_{\text{2}}\text{ max}$ protocol was considered one limitation of work-load setting for 7-by-1.

**Table 1:** Participant characteristics.

	Comparison group (*n* = 13)	7-by-1 HIT (*n* = 40)	5-by-1 HIT (*n* = 136)
Gender (men/women)	4/9	20/20	64/72
Age (years)	31 ± 11 (20–51)	37 ± 10 (20–53)	36 ± 9 (18–50)
Height (m)	1.66 ± 0.09 (1.52–1.81)	1.72 ± 0.09 (1.53–1.94)	1.72 ± 0.09 (1.50–2.01)
Body mass (kg)	93.1 ± 18.0 (68.6–130.5)	92.6 ± 17.5 (63.5–138.8)	95.1 ± 15.2 (64.0–136.4)
BMI (kg m^−2^)	33.4 ± 5.0 (27.5–41.4)	31.0 ± 4.2 (27.0–45.5)	32.2 ± 4.1 (26.5–48.1)
IPAQ score	305 ± 150 (118–578)	362 ± 157 (73–594)	313 ± 188 (0–597)
Baseline ${\dot{\text{V}}\text{O}}_{\text{2}}\text{ max}$ (mL kg^−1^ min^−1^)	24.1 ± 5.5 (13.2–32.0)	28.8 ± 7.0 (17.3–46.9)	27.2 ± 5.2 (15.8–44.6)
Systolic blood pressure (mmHg)	120 ± 9 (107–138)	124 ± 12 (106–161)	124 ± 11 (99–168)
Diastolic blood pressure (mmHg)	76 ± 9 (66–92)	78 ± 8 (67–105)	80 ± 10 (59–106)
Mean arterial pressure (mmHg)	91 ± 8 (80–104)	94 ± 9 (80–124)	95 ± 9 (74–127)
Log HOMA-IR	0.34 ± 0.17 (−0.06 to 0.51)	0.27 ± 0.24 (−0.27 to 0.92)	0.30 ± 0.26 (−0.46 to 0.86)

*Values shown are mean ± SD and range*.

*BMI, body mass index; IPAQ, International Physical Activity Questionnaire; $\dot{V}O_{2}\text{ max}$, maximal aerobic capacity; HOMA-IR, homeostatic model assessment of insulin resistance; HIT, high-intensity interval training*.

*Based on the study monitoring process, allocation of the participants to the HIT groups was sequential. The comparison group was utilized to examine test–retest performance over the study duration and not to adjust the HIT intervention responses. Direct physical activity monitoring was utilized to better link the HIT training directly to the changes in health biomarkers*.

### HIT—Protocol 2 (“5-by-1”)

The decision was made to use a higher intensity protocol, while subjects who had started the 7-by-1 HIT protocol completed the protocol and underwent a full clinical assessment (as the protocol may still have had benefits on IR). A further 136 participants completed baseline visits, HIT, and the post-HIT assessment [*n* = 64 men/72 women; age: 36 (18–50) years; BMI 32.2 (26.6–48.0) kg m^−2^] for a new higher intensity lower volume (5-by-1) HIT protocol (Table 1). The exercise training was fully supervised and consisted of three cycling sessions per week for 6 weeks. All sessions began with a 2-min warm-up at 50 W followed by five sets of 1 min high-intensity cycling work with 90 s recovery between sets with the exception of week 1 where three sets per session were performed in sessions 2 and 3. Work-load was determined in session 1 of week 1, where participants were asked to perform a 2-min warm-up at 50 W followed by 1-min bouts of exercise with 90 s recovery. Exercise started at 85% of the work required to elicit ${\dot{\text{V}}\text{O}}_{\text{2}}\text{ max}$ (Wmax), and increased by 10% (e.g., 95, 105%, etc.) until the participant was unable to complete a full 1-min bout. Intensity for the last bout participants could complete was used thereafter for training, with a 10% increase in intensity after 2 weeks. No adverse events or unintended effects were observed for this intervention.

### Non-Exercise Participants

Thirteen participants were allocated at random, within a center, to serve as a non-exercise comparison group [Table 1, *n* = 4 men/9 women; age: 31 (20–51) years; BMI 33.4 (27.5–41.4) kg m^−2^]. These participants underwent all screening and assessment procedures but did not participate in any training. Their data served to complement the short-term test–retest variability data collected in the intervention groups at the two baseline sessions with “test–retest” data covering the full duration of the study.

### Pre-Training Physiological Characterization

Participants were instructed to refrain from exercise for 3 days prior to their visit (baseline session 1) and from alcohol and caffeine for 1 day (fasting from ~09:00 p.m. and reporting to the laboratory 12 h later at ~09:00 a.m.). After 30 min supine rest, blood pressure (BP; Omron M2, Omron Healthcare, Kyoto, Japan) and resting heart rate (RHR) were measured, with mean arterial pressure (MAP) calculated as: 2/3 diastolic blood pressure + 1/3 systolic blood pressure. BP and RHR were determined as the average of three consecutive measurements. A blood sample was taken from a dorsal hand vein for the assessment of IR *via* the homeostatic model assessment (HOMA). Blood was immediately analyzed for glucose concentration (YSI 2300 STAT Plus glucose analyzer, Yellow Springs Inc., OH, USA) and aliquoted in to lithium heparin spray-coated vacutainers (Becton Dickinson, NJ, USA) and centrifuged at 2,000 *g* for 10 min at 4°C to yield plasma. Plasma was stored at −80°C and shipped for centralized analysis of insulin levels by a “high-sensitivity” ELISA (K6219, Dako Sweden AB, Stockholm) according to manufacturer’s instruction. HOMA-IR was calculated using the standard equation of [glucose (mmol/l) × insulin (mU/l)]/22.5 (23).

A ${\dot{\text{V}}\text{O}}_{\text{2}}\text{ max}$ test was then conducted using a cycle ergometer (Lode Corival/Excalibur Sport) and a continuous ramp protocol. After a 5-min warm-up at 50 W, the work rate was increased by 1 W every 4 s. Participants were instructed to cycle to volitional exhaustion. For the duration of the test, expired air was analyzed using an inline gas analyzer (e.g., Metamax 3B, Cortex, Leipzig, Germany; Vmax N29, Sensormedics, Anaheim, CA, USA; COSMED, Rome, Italy) with HR continuously monitored. ${\dot{\text{V}}\text{O}}_{\text{2}}\text{ max}$ was estimated as the highest value obtained in a 15-breath rolling average and a test was deemed valid when the participants achieved two of the following three criteria: (i) volitional exhaustion and/or no longer able to maintain a pedal rate of 50 revolutions per minute despite strong verbal encouragement, (ii) heart rate within 10 beats min^−1^ of age-predicted maximum, and (iii) respiratory exchange ratio (RER) ≥1.10. These criteria were met in all but one test, which was excluded from analysis of ${\dot{\text{V}}\text{O}}_{\text{2}}\text{ max}$. To assess the reproducibility of this ${\dot{\text{V}}\text{O}}_{\text{2}}\text{ max}$ test, the assessment was repeated 7 days later at baseline session 2 (as well as across 6 weeks in the non-training group). The coefficient of variation (CV) for repeated measurements for ${\dot{\text{V}}\text{O}}_{\text{2}}\text{ max}$ was 4.4%. As group mean ${\dot{\text{V}}\text{O}}_{\text{2}}\text{ max}$ was not different for visits 1 and 2 (2.59 ± 0.60 vs. 2.59 ± 0.63 L min^−1^, respectively) the mean of the two visits was taken as the subjects’ baseline value that reduces the influence of technical and biological variation and so should provide a better estimate of baseline ${\dot{\text{V}}\text{O}}_{\text{2}}\text{ max}$. At 72–96 h after the last exercise training session, participants underwent a third study day, identical to visit 1.

### PA and Post-Training Monitoring

Physical activity was monitored using Actiheart devices (CamNTech, Cambridge, UK), a chest-worn monitor that records heart rate and movement *via* an accelerometer. The device senses the frequency and intensity of torso movements and has been shown to be comparable to doubly labeled water for measuring energy expenditure (24). Activity data were obtained for 7 days prior to study visit 1, prior to study visit 2, during week 3 or 4 of HIT, and prior to study visit 4 (during the detraining period). Participants were instructed to wear the Actiheart device at all times during the monitoring periods (using waterproof Actiheart chest strap or using standard ECG electrodes). Participants using the ECG electrodes were instructed to place one electrode at the site of the fourth intercostal with the second electrode ~10 cm to the left (equivalent to V1 and V4 on a 12-lead ECG). These participants were instructed to wear the monitor at all times with the exception of a very short period each day when they were instructed to thoroughly wash and dry the skin under the electrodes in order to minimize the risk of contact dermatitis or other skin irritation. After completion of the exercise training intervention, participants were asked to return to their habitual PA levels for 3 weeks (confirmed by Actiheart) and then a fourth study day, identical to visit 3, was carried out.

### Data Processing and Statistical Analysis

To bench mark these HIT protocols with literature values, a robust post-training group average increase in ${\dot{\text{V}}\text{O}}_{\text{2}}\text{ max}$ had to be evident. Power analysis indicated that >29 participants would be required to detect a 4% difference between pre- and post-training ${\dot{\text{V}}\text{O}}_{\text{2}}\text{ max}$ with a power of 95% and alpha = 0.05, based on a CV of 5.7%. To detect a difference of 4% between men and women, for change in ${\dot{\text{V}}\text{O}}_{\text{2}}\text{ max}$, with alpha = 0.05 and a power of 95%, >53 participants were required. Thus, the analysis was powered for primary statistical analysis presented in this paper.

Statistical analysis was performed using SPSS statistical software (version 20.0, SPSS Inc., Chicago, IL, USA). Data were either tested for normality using the Shapiro–Wilks test and analyzed with non-parametric tests or log transformed. Differences between pre- and post-training values were evaluated using paired sample *t*-tests [*n* = 40 and *n* = 136 for ${\dot{\text{V}}\text{O}}_{\text{2}}\text{ max}$, and *n* = 36 and *n* = 133 for HOMA-IR for 7-by-1 and 5-by-1 protocols, respectively (reflecting any missing values)]. Effect size was quantified using Cohen’s *d* (25). Gender differences in training response were analyzed using independent sample *t*-tests. Bivariate correlations were assessed using Pearson’s correlation coefficient. Repeated measures ANOVAs with *post hoc* Bonferroni tests for multiple comparisons were used to assess retention of training effects following 5-by-1 HIT for those participants who completed study visit 4 (Figure 1). All data are presented as mean ± SD unless stated otherwise.

Quantification of inter-individual responses to training, corrected for estimates of random variation (technical/day-to-day biological) was performed according to the procedures proposed by Hopkins (26). SD for individual responses (SD_IR_) were calculated by taking the square root of the difference between the squares of the SD of the training effect (SD_exp_) and the SD of either the double baseline measurement (for variables measured twice before training) or the SD of the repeated measures carried out in the comparison group (SD_con_). In addition, paired sample *t*-tests were performed to determine differences between SD_exp_ and SD_con_, and Levene’s test was performed to determine differences between the SD_exp_ for 5-by-1 HIT and the SD_exp_ for an earlier study that utilized high volume combined aerobic/resistance training [STRRIDE AT/RT study (27)].

Actiheart data were scanned for missing values using a heuristic code in R, and data accepted only when ≥80% of minute-by-minute activity data were available for a 24-h recording period. Furthermore, at least 4 days of valid data had to be available for a participant to be included in the group analysis (leaving *n* = 58 for 5-by-1 HIT). Mean daily energy expenditure (METs) for each of the four measurement periods was calculated, and standard thresholds were used to determine the percentage of time engaged in activity within predetermined intensity zones (sedentary: <1.5 METs; light ≥1.5 < 3 METs; moderate ≥3 < 6 METs; vigorous ≥6 < 10.2 METs; very vigorous ≥10.2 METs). Reliability of the Actiheart data, using this data selection criteria, was excellent (*R*^2^ = 0.87 for the repeated baseline measure; CV = 4.8%). The mean values obtained prior to study visits 1 and 2 were used as the baseline values.

## Results

### Training Responses

Following 6 weeks of 7-by-1 HIT, there were modest improvements in mean ${\dot{\text{V}}\text{O}}_{\text{2}}\text{ max}$ (+6.2%, 95% CI: 3.5–8.9%, *p* < 0.001). This equates to a moderate effect size, i.e., Cohen’s *d* = 0.71 (95% CI = 0.25–1.16) for the primary outcome. As expected, Wmax (5.3%; *p* < 0.001) was also increased by 7-by-1 HIT, but no other outcomes were significantly altered. For the control group that undertook two assessments 6 weeks apart, we observed no significant changes between baseline assessment and reassessment 6 weeks later in any parameter.

**Figure 2:**
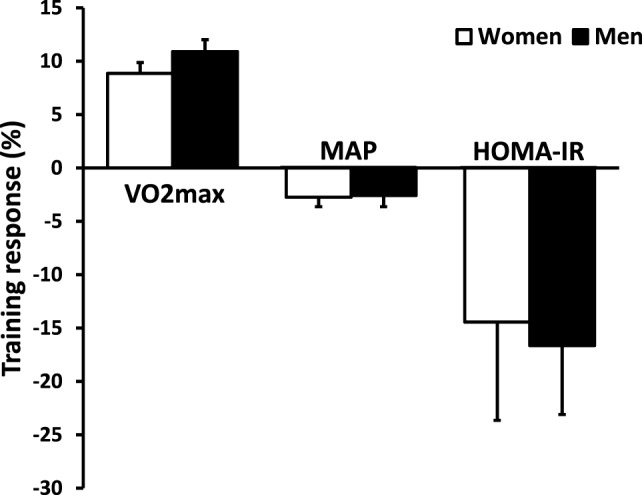
An analysis of the training response to 5-by-1 HIT for $\dot{\text{V}}{\text{O}}_{2}\text{ max}$, MAP and HOMA-IR in men and women. Inter-participant variability in response to HIT is large for both men and women, but on average both genders respond to a similar extent. Abbreviations: $\dot{\text{V}}{\text{O}}_{2}\text{ max}$, maximal aerobic capacity; MAP, mean arterial pressure; HOMA-IR, homeostatic model assessment of insulin resistance; HIT, high-intensity interval training.

Following 6 weeks of the more time-efficient 5-by-1 HIT protocol, greater changes were observed for ${\dot{\text{V}}\text{O}}_{\text{2}}\text{ max}$ (+10.0%, 95% CI: 8.4–11.6%; *p* < 0.001) presenting a larger and less variable size effect (Cohen’s *d* = 1.24, 95% CI = 0.97–1.50) (Table 2). The increase in ${\dot{\text{V}}\text{O}}_{\text{2}}\text{ max}$ with 5-by-1 HIT was also greater than we observed with 7-by-1 HIT (*p* < 0.05) supporting our interim analysis and decision to discontinue that protocol. The *absolute* increase in ${\dot{\text{V}}\text{O}}_{\text{2}}\text{ max}$ with 5-by-1 HIT was significantly higher for men (0.32 ± 0.3 L min^−1^) compared to women (0.19 ± 0.2 L min^−1^; *p* < 0.001) as expected, but the relative benefits were not (Figure 2). Furthermore, 5-by-1 HIT yielded reductions in MAP (2.8%; *p* < 0.001) and HOMA-IR (16%; *p* < 0.01) (Table 2). Similarly, no significant gender differences were apparent for the relative training response for MAP or HOMA-IR (Figure 2). Thus, we found no evidence that HIT-induced physiological adaptations were subject to gender-related dimorphism.

**Table 2:** Mean physiological changes following 6 weeks of time-efficient high-intensity cycle-training.

	7-by-1 HIT (*n* = 40)			5-by-1 HIT (*n* = 136)		
	Baseline	Post-HIT	*p*-Value	Baseline	Post-HIT	*p*-Value
BMI (kg m^−2^)	31.0 ± 4.2	30.8 ± 4.2	0.138	32.2 ± 4.1	32.1 ± 4.2	0.98
Body mass (kg)	92.4 ± 17.3	92.0 ± 17.8	0.210	95.1 ± 15.2	95.1 ± 15.4	0.98
$\dot{\text{V}}{\text{O}}_{2}\text{ max}$ (L min^−1^)	2.61 ± 0.60	2.77 ± 0.68	0.00005	2.59 ± 0.62	2.85 ± 0.68	<1E−20
Wmax at $\dot{\text{V}}{\text{O}}_{2}\text{ max}$ (W)	189 ± 50	199 ± 51	0.001	198 ± 48	226 ± 53	<1E−23
SBP (mmHg)	124 ± 12	122 ± 11	0.169	124 ± 11	122 ± 12	0.007
DBP (mmHg)	78 ± 8	77 ± 8	0.281	80 ± 10	77 ± 10	0.0006
MAP (mmHg)	94 ± 9	92 ± 8	0.183	95 ± 9	92 ± 9	0.0001
Fasting glucose (mmol L^−1^)	4.56 ± 0.32	4.57 ± 0.40	0.855	4.63 ± 0.41	4.60 ± 0.45	0.61
Fasting insulin (pmol L^−1^)	10.6 ± 6.4	10.2 ± 6.8	0.494	11.3 ± 6.6	10.5 ± 6.7	0.005
Log HOMA-IR	0.27 ± 0.24	0.23 ± 0.30	0.187	0.30 ± 0.26	0.25 ± 0.27	0.004

*Values shown are mean ± SD*.

*BMI, body mass index; $\dot{V}O_{2}\text{ max}$, maximal aerobic capacity; Wmax, maximum power output; SBP, supine systolic blood pressure; DBP, supine diastolic blood pressure; MAP, supine mean arterial pressure; HOMA-IR, homeostatic model assessment of insulin resistance; HIT, high-intensity interval training*.

*Note that the intensity of each bout during the 7-by-1 protocol was 20–30% lower than during the 5-by-1 protocol, indicating that relying on supramaximal (from the perspective of aerobic capacity) is probably important for gains in aerobic capacity. Changes in a measure of peripheral insulin resistance (HOMA-IR) were more variable with 7-by-1 and not improved with the *n* = 40 sample size for the selected threshold for statistical significance*.

Actiheart-derived PA data demonstrated a small increase in PA energy expenditure during the 6-week intervention period of 5-by-1 HIT (mean 24-h activity level: 1.46 ± 0.38 vs. 1.50 ± 0.34 METs) equivalent to an increase of ~400 MET min week^−1^ (*p* < 0.05). This increase was accounted for by increases in the percentage of time spent performing vigorous (0.30 ± 0.47 vs. 0.43 ± 0.43%, *p* < 0.001) and very vigorous activities (0.02 ± 0.09 vs. 0.07 ± 0.16%, *p* < 0.001), i.e., the 18 HIT sessions. No change was observed in the percentage of time spent in sedentary (74.1 ± 16.3 vs. 74.0 ± 14.9%), light (18.0 ± 9.1 vs. 17.8 ± 8.6%), and moderate activity zones (7.6 ± 8.2 vs. 8.2 ± 7.0%). Thus, carrying out 5-by-1 HIT did not alter PA behavior out with the trial.

### Comparison of Inter-Individual Variability between HIT and High-Volume Training

**Figure 3:**
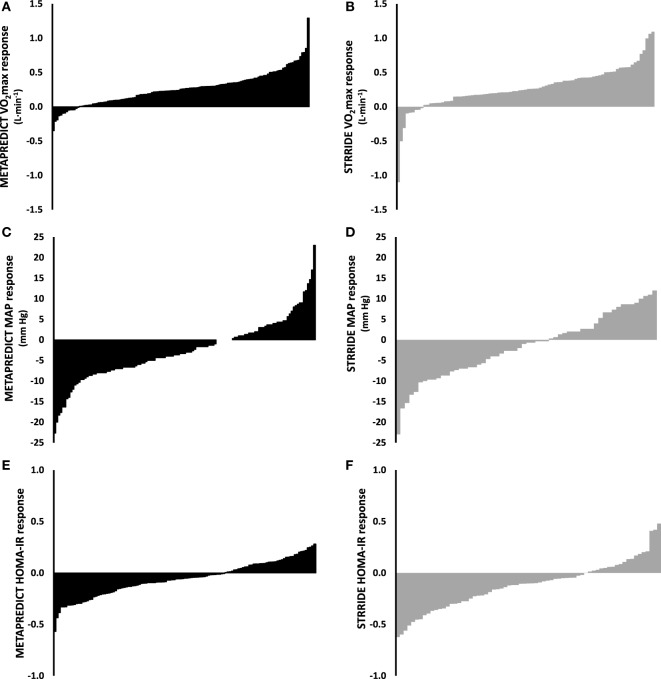
Comparison of the inter-individual variability to exercise training contrasting short-term high-intensity training with longer-term high-volume submaximal training. The training response to 6-weeks 5-by-1 high-intensity interval training [**(A,C,E)**; black bars] and our previously published 8-month STRRIDE AT and AT/RT exercise training study [**(B,D,F)**; gray bars] for $\dot{\text{V}}{\text{O}}_{2}\text{ max}$, MAP, and HOMA-IR. Training-induced changes in both ${\dot{\text{V}}\text{O}}_{\text{2}}\text{ max}$ **(A,B)**, MAP **(C,D)**, and HOMA-IR **(E,F)** vary considerably in both studies and to a similar extent. Abbreviations: AT, aerobic training; RT, resistance training; ${\dot{\text{V}}\text{O}}_{\text{2}}\text{ max}$, maximal aerobic capacity; MAP, mean arterial pressure; HOMA-IR, homeostatic model assessment of insulin resistance.

Inter-individual variability (Figure 3) in training responses reflects the fact that there are genuine low and high responders for major physiological traits, following any type of exercise training program. This variability will be partly due to random contributions from technical and day-to-day biological variation, and partly due to genetic differences between individuals (28). For 5-by-1 HIT, change in ${\dot{\text{V}}\text{O}}_{\text{2}}\text{ max}$ $\left(\Delta {\dot{\text{V}}\text{O}}_{\text{2}}\text{ max}\right)$ was not correlated to baseline ${\dot{\text{V}}\text{O}}_{\text{2}}\text{ max}$ (*R*^2^ = 0.01, NS) such that low baseline aerobic capacity was not associated with a greater training response nor *vice versa*. By contrast, ΔMAP was negatively correlated to baseline MAP (*R*^2^ = 0.18, *p* < 0.001), and Δ log HOMA-IR was negatively correlated to baseline log HOMA-IR (*R*^2^ = 0.07, *p* < 0.01). In a population sample that had a range of blood pressure and log HOMA-IR spanning normal to above normal (74 to 127 mmHg and −0.46 to 0.86, respectively), such a correlation is expected as both parameters are regulated toward a physiologically “normal” value. Nevertheless, on an individual basis, this analysis, such as others before it, demonstrates that baseline physiological measures are not, on their own, useful at predicting the health biomarker outcomes of an exercise training regime, indicating that more sophisticated strategies will be required to fulfill such an aim (29).

To contrast the variation observed in response to 5-by-1 HIT with traditional higher volume exercise training (Figure 3) (30), we estimated the “added” variation caused by the training intervention (SD_IR_), over and above the random variation by comparing the variability in repeated measures at baseline [or in a control group (SD_con_)] with the observed variability in response to the training intervention (SD_exp_). For 5-by-1 HIT, the SD_con_ for ${\dot{\text{V}}\text{O}}_{\text{2}}\text{ max}$ (visit 1 vs. visit 2; 112 ± 94 mL) was lower than SD_exp_ (visit 2 vs. visit 3; 204 ± 150 mL; *p* < 0.001). For 5-by-1 HIT, the SD_IR_ was calculated to be 170 mL (95% CI: 23–311 mL). In standardized units, the magnitude of the effect for the individual responses was large (0.67; 95% CI: 0.11–1.22). For ${\dot{\text{V}}\text{O}}_{\text{2}}\text{ max}$, the SD_exp_ from our previously published data (30) was not significantly different from 5-by-1 HIT (204 vs. 234 mL O_2_). Based on this analysis, 95% of people performing 5-by-1 HIT can be expected to have a “true” response for ${\dot{\text{V}}\text{O}}_{\text{2}}\text{ max}$ between −79 and +587 mL O_2_. Similarly, for MAP, SD_exp_ exceeded SD_con_ for 5-by-1 HIT (4.2 vs. 2.6 mmHg, respectively) resulting in an SD_IR_ of 3.3 mmHg (95% CI: 0.3–6.3 mmHg). This is also a large effect in standardized units (−1.32; 95% CI: −2.50 to 0.13) and indicates that for 5-by-1 HIT, 95% of people can be expected to have a response for MAP within −9.0 and +4.0 mmHg, i.e., considerable inter-individual variability in response to HIT. Despite the extreme differences in the format (volume and intensity) of exercise training between 5-by-1 HIT and STRRIDE AT/RT (30), no significant differences were observed between their respective SD_exp_ for blood pressure (MAP: 4.2 vs. 4.5 mmHg) (Figure 3).

**Figure 4:**
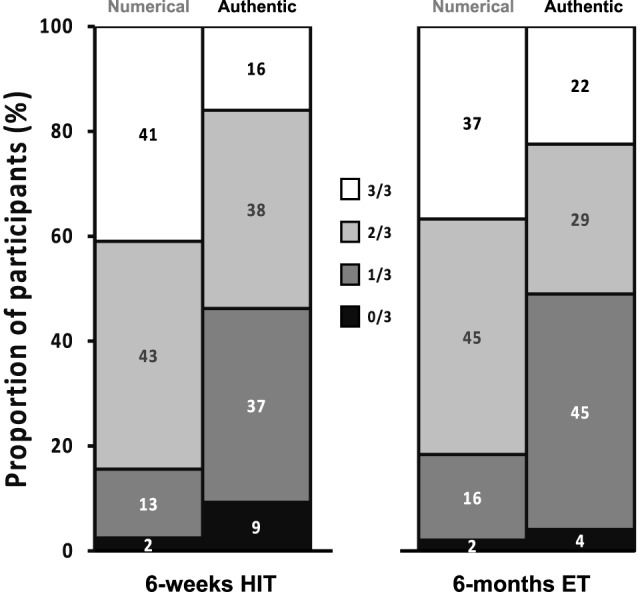
Presentation of the responder frequency for the three main clinical biomarkers considered in this study [high-intensity interval training (HIT)] and comparison with our previously published endurance training (ET) study. Each individual was assessed for improvement in $\dot{\text{V}}{\text{O}}_{2}\text{ max}$, mean arterial pressure, or HOMA-IR, greater than the laboratory error, and the percentile frequency of 0, 1, 2, or 3 from three improvements was calculated. For sake of comparison, this is plotted side-by-side with the percentile frequency of 0, 1, 2, or 3 gains based on numerical improvements (a criteria that would be considered unreliable by most). Approximately 40% of subjects demonstrate improvement in only one health biomarker, while between 4 and 9% demonstrate no reliable improvement in any.

The fact that the pattern of variability in response for ${\dot{\text{V}}\text{O}}_{\text{2}}\text{ max}$, MAP, and HOMA-IR (three key biomarkers for cardio-metabolic health) to 6 weeks of 5-by-1 HIT is not different from that observed in an 6-month high-volume aerobic/resistance training intervention suggests that inter-individual variability in responses to training is not dependent on exercise mode, exercise-session duration, total volume, or the duration of the intervention, but rather depends on genetics, epigenetics, and other biological factors (28). One important practical consideration is the proportions of subjects that demonstrates “real” improvements in each of the main health biomarkers. To address such a question, we counted the frequency of people with 0, 1, 2, or 3 positive changes in ${\dot{\text{V}}\text{O}}_{\text{2}}\text{ max}$, MAP, and HOMA-IR defined as an improvement over and above technical error for that physiological parameter. As can be observed in Figure 4, whether one considers the frequency of observing a numerical improvement (unreliable) or a gain that is greater than the normal technical error for the test, ~50% of subjects improve at least two of the three health biomarkers following 6 months endurance training or 6 weeks of 5-by-1 HIT.

### Physiological Changes during Detraining

**Figure 5:**
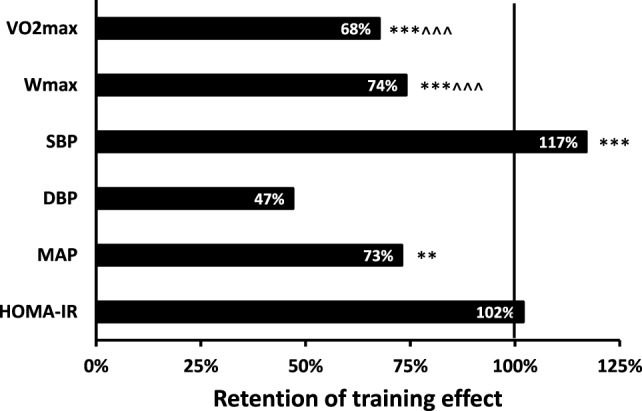
Presentation of the average retention of the training-induced changes observed 3 weeks after cessation of 5-by-1 high-intensity interval training. A value of 100% represents the training effect and a value of 0% indicates that the training effect is lost 3 weeks after training (under sedentary conditions). Significant differences from baseline: **p < 0.01, ***p < 0.001. Significant differences from post-training: ^^^^^*p* < 0.001. Abbreviations: $\dot{\text{V}}{\text{O}}_{2}\text{ max}$, maximal aerobic capacity; Wmax, maximal power output; SBP, systolic blood pressure; DBP, diastolic blood pressure; MAP, mean arterial pressure; HOMA-IR, homeostatic model assessment of insulin resistance.

As a secondary objective, we evaluated the status of training-induced changes in physiological parameters, from 6 weeks of 5-by-1 HIT, during a 3-week period where subjects returned to their baseline sedentary lifestyle (Figure 5). Seven participants (~5%) were lost to follow-up during this period. Actiheart-derived PA measures confirmed that subjects had returned to baseline sedentary behavior (1.48 ± 0.37 METs). ${\dot{\text{V}}\text{O}}_{\text{2}}\text{ max}$ tended toward pre-training levels (32% reversal; *p* < 0.001) after 3 weeks of Actiheart-verified sedentary behavior, yet remained elevated above pre-training values (*p* < 0.001). The reversal of exercise induced changes in MAP following detraining were partial, whereas the HIT-induced changes in HOMA-IR were fully retained during this 3-week period, consistent with some earlier pilot metabolic protein data (31).

${\dot{\text{V}}\text{O}}_{\text{2}}\text{ max}$, MAP, and HOMA-IR each displayed negative correlations between the changes following 6 weeks of 5-by-1 HIT and changes following 3 weeks of detraining (${\dot{\text{V}}\text{O}}_{\text{2}}\text{ max}$: *R*^2^ = 0.12, *p* < 0.001; MAP: *R*^2^ = 0.30, *p* < 0.001; HOMA-IR: *R*^2^ = 0.15, *p* < 0.001); i.e., high-responding participants tended to lose a greater amount of their training gains compared to low responders, which is logical and further supports that the determinations of training-induced changes were biological in origin. SD_exp_ for ${\dot{\text{V}}\text{O}}_{\text{2}}\text{ max}$ for detraining effects exceeded SD_con_ (178 vs. 112 mL O_2_, respectively), resulting in an SD_IR_ of 138 mL O_2_ (95% CI: 12–264 mL O_2_). Similarly, SD_exp_ for MAP for detraining effects exceeded SD_con_ (4.2 vs. 2.6 mmHg, respectively), resulting in an SD_IR_ of 3.3 mmHg (95% CI: 0.3–6.3 mmHg). This suggests the existence of low and high responders for retention of training effects. However, the amplified effect of technical and day-to-day biological variability on delta-scores compared to absolute scores limits our ability to draw conclusions on whether variability in the responses to training and detraining are strongly linked.

## Discussion

In this study, we adapted an exercise protocol used by Ready et al. (19) so that it was practical to implement using a standard electrically braked cycle ergometer and involve a total time-commitment of <15 min. We then demonstrated that, on average, 6 weeks of this 5-by-1 protocol was efficacious at reducing blood pressure and peripheral IR, while increasing aerobic capacity and all to an extent identical to high-volume exercise training carried out over 6 months (8). These observations enable us to claim that time-efficient exercise (<45 min week^−1^) can reduce type 2 diabetes and cardiovascular disease risk factors in overweight men and women. If this exercise behavior was maintained, it should yield long-term health benefits (32) with a fraction of the required time-commitment associated with the recommendations by current public health guidelines (6).

Ready et al. demonstrated over 35 years ago, in Ontario, Canada, that 10 1-min intervals at a work-load equating to ~110% ${\dot{\text{V}}\text{O}}_{\text{2}}\text{ max}$, interspersed with 1-min recovery intervals, yielded ~8% gains in ${\dot{\text{V}}\text{O}}_{\text{2}}\text{ max}$. Despite this observation, awareness of the potential utility of HIT has only emerged in recent years. Recently, Little et al. using the same protocol as Ready et al., found improved glycemic control in a small group of people with type 2 diabetes (22) and we now demonstrate that, in a large group of subjects at-risk for developing type 2 diabetes, HIT can improve HOMA-IR with a more time-efficient version of this protocol. 5-by-1 HIT, relying on 50% fewer sprints than Ready et al. (19), but at a greater intensity (~125 vs. ~110% ${\dot{\text{V}}\text{O}}_{\text{2}}\text{ max}$), produced an equally robust increase in ${\dot{\text{V}}\text{O}}_{\text{2}}\text{ max}$ (~10%) and reductions in HOMA-IR (~16%). We also noted that 6 weeks of 5-by-1 HIT has a mean effect for ${\dot{\text{V}}\text{O}}_{\text{2}}\text{ max}$ comparable with 6 months of traditional high-volume time-consuming exercise training, indicating that time-efficient HIT can match the efficacy of traditional exercise training paradigms for the present health biomarkers, as proposed from earlier pilot studies (33).

This study is the first to attribute improvements in health biomarkers to the HIT sessions *per se*, as we show that performing HIT did not result in alterations in PA out with the supervised training sessions. The lack of “extra-curricular” changes in PA is consistent with observations made during studies involving long-term high-volume exercise training (34). The estimation of energy expenditure using Actiheart monitors enabled us to present the HIT intervention protocol in units consistent with public health orientated measures of PA. The Actiheart device appears sufficiently sensitive to pick up high-intensity exercise performed during the HIT sessions, providing reliable free-living data on both total PA levels and time spent performing activities of different intensities. We found that subjects performing 5-by-1 HIT had an increase in energy expenditure of ~400 MET min week^−1^, consistent with the lower end of the current US Department of Health “time orientated” recommendations for PA (500–1,000 MET min week^−1^). Thus, we were able to demonstrate that it is possible to reach these MET targets in a highly time-efficient manner. There were, however, technical limitations of the Actiheart monitoring, namely the devices produced acceptable data for less than half of our participants (reflecting obvious and periodic loss of signal). The participants received clear instructions on how to correctly wear the activity monitor during free-living conditions, and we do not know what caused the loss of signal and further research is needed to make continuous PA monitoring more reliable.

Importantly, we found that response variability in response to 6 weeks of 5-by-1 HIT exceeds technical and day-to-day biological variability for aerobic capacity and blood pressure and that this variability was similar that observed following 6 months of high-volume exercise training (9, 35). We observed, for the present three sessions per week training program, a rate of non-responders for ${\dot{\text{V}}\text{O}}_{\text{2}}\text{ max}$ (~15–20%) comparable to many other high volume training programs, involving thousands of volunteers typically training 4–5 days week^−1^ (15, 36–39). Recently, it has been claimed that non-responders for ${\dot{\text{V}}\text{O}}_{\text{2}}\text{ max}$ “do not exist” (40). This conclusion was based on “under-training”, then re-training four groups of 10 subjects with differing frequencies of training per week. The study used a spuriously and low value for the ${\dot{\text{V}}\text{O}}_{\text{2}}\text{ max}$ testing variation, i.e., the Wmax error, and failed to consider that this “error” applies to both the pre-test and post-test values, seriously undermining the validity of the study. In addition, they could not replicate in *phase one* of their “study,” the known non-response rate for ${\dot{\text{V}}\text{O}}_{\text{2}}\text{ max}$ seen in much larger studies using their 4–5 days week^−1^ training protocol (15, 36–39), suggesting some form of recruitment bias. Careful consideration of their data, claims, and an appropriate cutoff value for measurement variance indicates that the conclusions reached (40) are misleading. Thus, large and robust studies have found that physiological responses are heterogeneous to every type of exercise training program. Indeed, we present a meta-analysis of the genuine response frequencies for our three clinically relevant health biomarkers, ${\dot{\text{V}}\text{O}}_{\text{2}}\text{ max}$, BP, and HOMA-IR (Figure 4), demonstrating that at least 50% of the population can expect to be a non-responder for one of these biomarkers. This is somewhat in agreement with the efficacy noted in the long-term diabetes prevention studies (3–5), where type 2 diabetes risk is reduce but not eliminated.

We can, therefore, conclude that the present 5-by-1 HIT protocol is consistent with other exercise programs, and that it is *on average* sufficient to yield improvements in cardiovascular and metabolic parameters in both men and women. Weston et al. (41) recently conducted a meta-analysis and concluded that improvements in the ${\dot{\text{V}}\text{O}}_{\text{2}}\text{ max}$ of sedentary males (10.0%; 90% CI: 4.9–15.1%) was greater than for sedentary females (7.3%; 2.5–12.1%). We would argue that an accurate estimation of the size effect of HIT using meta-analysis methodology and numerous very disparate small studies is not robust due to large variations in protocol design. While the large confidence intervals presented by Weston et al. were indicative of a high level of uncertainty in their analysis, this study relied on a large cohort of men and women undertaking an identical training program and measurement protocol, and found gains in ${\dot{\text{V}}\text{O}}_{\text{2}}\text{ max}$ were in fact comparable in men and women. The same conclusion can be reached regarding blood pressure and fasting IR.

Various HIT-like protocols have been utilized in patient groups to promote rehabilitation and control risk factors for disease (42–44). In fact, many HIT-type protocols have been utilized safely in cardiac patients for many years (45). In this study, we did not observe any adverse clinical events in a group of sedentary participants with risk factors for cardiovascular and metabolic disease. However, we do not have the required size or duration of follow-up to make recommendations on safety (or disease prevention), as such an analysis will require thousands of participants (as serious acute clinical events are rare during exercise training). Nevertheless, given that the 5-by-1 protocol yields a PA MET “score” comparable to current PA targets, is equally effective at improving aerobic capacity and reducing IR, it would seem reasonable to conclude that it can emerge as an effective alternative to high-volume time-consuming aerobic exercise training. This is particularly true as the majority of the adult population do not meet the lower-intensity time-orientated targets and, thus, do not gain some of the benefits of an active lifestyle. Thus 5-by-1 HIT could substantially reduce the incidence or progression-rates of type 2 diabetes similar to previous long-term lifestyle interventions (3–5). Notably, the improvement in HOMA-IR following 6 weeks HIT is comparable in magnitude to 2 years of calorie restriction (46) supporting the idea that increased levels of PA *via* HIT could directly contribute to the prevention of type 2 diabetes more rapidly that other types of intervention.

## Ethics Statement

This study was approved by local ethics committees at all sites [University of Nottingham (D8122011 BMS), Karolinska Institutet (2012/753-31/2), the University of Copenhagen (H-3-2012-024), the University of Las Palmas de Gran Canaria (CEIH-2012-02), and Loughborough University (12/EM/0223)] and complied with the 2008 Declaration of Helsinki.

## Author Contributions

The META-PREDICT application was written in 2010 by JT, and awarded in 2011 with written contributions from PA and OR. BP, NV, PA, TG, BK, WK, JT, and OR contributed to the design of the study. All authors contributed to data acquisition (BP, BK, ML, JP-G, TG, and OR), data analysis (BP, BK, OR, RB, DM, JT, and NV), or interpretation of data (BP, BK, NV, OR, RB, PA, WK, and JT). JT, BP, WK, RB, OR, and NV drafted the manuscript for publication, while all authors contributed to critically reviewing the manuscript for intellectual content. All authors gave final written approval of the manuscript for publication and agreed to be accountable for the accuracy and integrity of the data.

## Conflict of Interest Statement

JT, TG, OR, BP, and NV are shareholders in XRGenomics LTD. The authors have no further interests to declare and the present article does not represent any protected information.
